# Inhaled glucocorticoid-induced metabolome changes in asthma

**DOI:** 10.1530/EJE-21-0912

**Published:** 2022-07-04

**Authors:** Peter Daley-Yates, Brian Keppler, Noushin Brealey, Shaila Shabbir, Dave Singh, Neil Barnes

**Affiliations:** 1 Clinical Pharmacology and Experimental Medicine, GSK, Uxbridge, UK; 2 Discovery and Translational Sciences, Metabolon Inc., Morrisville, North Carolina, USA; 3 Medicines Research Centre, GSK, Stevenage, UK; 4 Medicines Evaluation Unit, University of Manchester, Manchester University NHS Foundation Trust, Manchester, UK; 5 Global Medical Franchise, GSK, Brentford, UK; 6 William Harvey Institute, Bart’s and the London School of Medicine and Dentistry, London, UK

## Abstract

**Objective:**

The aim of this study was toidentify dose-related systemic effects of inhaled glucocorticoids (GCs) on the global metabolome.

**Design and methods:**

Metabolomics/lipidomic analysis from plasma was obtained from 54 subjects receiving weekly escalating doses (µg/day) of fluticasone furoate (FF; 25, 100, 200, 400 and 800), fluticasone propionate (FP; 50, 200, 500, 1000 and 2000), budesonide (BUD; 100, 400, 800, 1600 and 3200) or placebo. Samples (pre- and post-dose) were analysed using ultrahigh-performance liquid chromatography-tandem mass spectroscopy and liquid chromatography-mass spectrometry. Ions were matched to library standards for identification and quantification. Statistical analysis involved repeated measures ANOVA, cross-over model, random forest and principal component analysis using log-transformed data.

**Results:**

Quantifiable metabolites (1971) had few significant changes (% increases/decreases; *P* < 0.05) vs placebo: FF 1.34 (0.42/0.92), FP 1.95 (0.41/1.54) and BUD 2.05 (0.60/1.45). Therapeutic doses had fewer changes: FF 0.96 (0.36/0.61), FP 1.66 (0.44/1.22) and BUD 1.45 (0.56/0.90). At highest/supratherapeutic doses, changes were qualitatively similar: reduced adrenal steroids, particularly glucuronide metabolites of cortisol and cortisone and pregnenolone metabolite DHEA-S; increased amino acids and glycolytic intermediates; decreased fatty acid β-oxidation and branched-chain amino acids. Notable qualitative differences were lowered dopamine metabolites (BUD) and secondary bile acid profiles (BUD/FF), suggesting CNS and gut microbiome effects.

**Conclusions:**

Dose-dependent metabolomic changes occurred with inhaled GCs but were seen predominately at highest/supratherapeutic doses, supporting the safety of low and mid therapeutic doses. At comparable therapeutic doses (FF 100, FP 500 and BUD 800 µg/day), FF had the least effect on the most sensitive markers (adrenal steroids) vs BUD and FP.

## Introduction

Standard of care for asthma includes anti-inflammatory inhaled glucocorticoids (GCs) and smooth muscle-relaxing bronchodilators (1). Low-dose inhaled GCs provide clinical benefit to most patients with asthma (1). This is reflected in management guidelines that recommend low-dose inhaled GCs in mild–moderate asthma, whereas high-dose inhaled GCs are recommended for more severe asthma (1). Chronic use of high-dose inhaled GCs can cause systemic adverse effects, including hypothalamus–pituitary–adrenal (HPA) axis dysfunction, effects on bone and muscle, energy metabolism, hyperglycaemia, skin thinning, bruising and psychiatric/behavioural disturbances (1, 2, 3). There is evidence that inhaled GCs can affect adrenal steroid pathways (4), but the association with pathways linked to other systemic effects has not been well studied. Furthermore, these effects have not been fully characterised at the molecular level; qualitative and quantitative differences between different GC molecules have not been investigated.

Traditionally, systemic effects of GCs have been assessed using HPA axis studies or studies of specific organ systems (5). Metabolomics is the systematic analysis of functional metabolites present in biological systems, including carbohydrates, amino acids, organic acids, nucleotides and lipids (6), offering an alternative method to measure the systemic effects of inhaled GCs. The metabolome is seen as the convergence of gene expression and environmental exposure and can provide an integrated measure of these effects (7, 8). Previously reported metabolomic analyses in asthma have focused on the impact of asthma severity and differences versus healthy subjects (8, 9, 10, 11, 12, 13, 14, 15), focused on differences vs healthy controls and with varying degrees of asthma severity (8). These studies identified molecules and pathways associated with asthma pathophysiology, including energy metabolism (8), redox status (9, 10), immunity (11, 12, 13), inflammation (13, 14), lipid metabolism (9, 11, 13, 14) and the tricarboxylic acid (TCA) cycle (15).

We previously described the dose–response for the bronchoprotective effects of three inhaled GCs in asthma, providing evidence that not all inhaled GCs are therapeutically similar. Fluticasone furoate (FF) offered more protection against airway hyperresponsiveness to adenosine-5’-monophosphate challenge, with less systemic activity than fluticasone propionate (FP) or budesonide (BUD) (16). Here, we provide a global metabolomic analysis from this study, comparing the systemic metabolic dose–responses for these compounds. The aim was to identify qualitative, quantitative and dose-related effects that arise via glucocorticoid receptor (GR) and non-GR-mediated actions.

## Subjects and methods

### Study design

Metabolomic analysis of the systemic effects of three inhaled GC molecules (FF, FP and BUD) was conducted as part of a randomised, escalating-dose, placebo-controlled, incomplete-block study in subjects with mild asthma (16) (ClinicalTrials.gov NCT02991859). The study was conducted at two centres in the UK and one in Germany.

**Figure 1:**
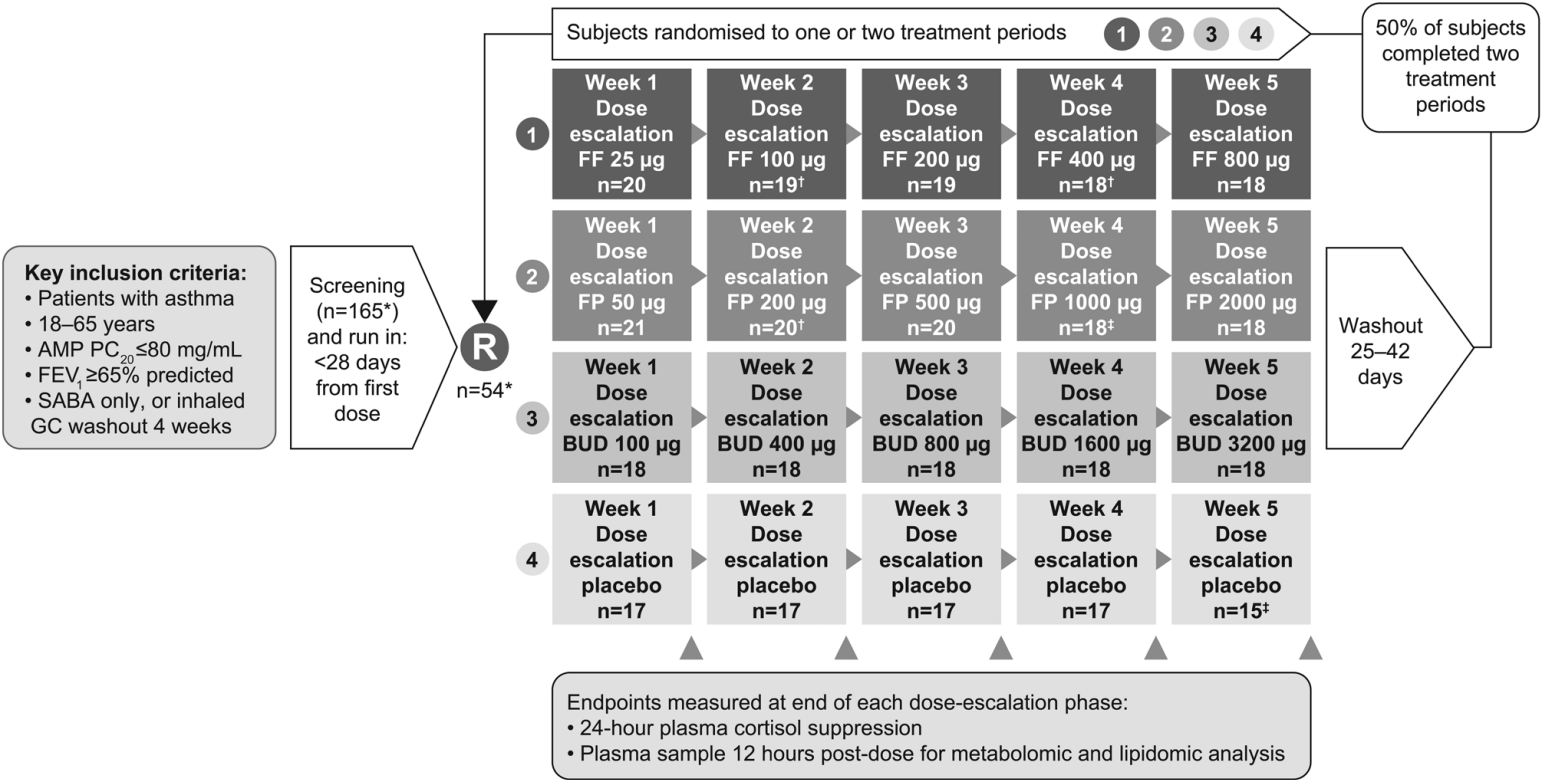
Study design. *165 subjects were screened; 56 subjects were enrolled; 108 subjects excluded (76 subjects did not meet eligibility criteria; 15 subjects withdrew consent, 1 subject withdrawn at investigator’s discretion; 18 subjects were excluded for ‘other’ reasons); 54 subjects were randomised; 2 subjects withdrew prior to randomisation. Pre-dose plasma samples for cortisol and metabolomic analysis were also taken before the start of each period. ^†^1 subject withdrawn. ^‡^2 subjects withdrawn/excluded. AMP PC_20_, provocative concentration of adenosine-5’-monophosphate resulting in a decline of ≥ 20% in FEV_1_; BUD, budesonide; FEV_1_, forced expiratory volume in 1 s; FF, fluticasone furoate; FP, fluticasone propionate; GCs, glucocorticoid; R, randomisation; SABA, short-acting beta-agonist.

Subjects consented and were randomised to undertake one or two of the four treatment periods (FF, FP, BUD or placebo) separated by a 25–42 day washout period (Fig. 1). Each treatment period lasted for 35 days and comprised five consecutive dose escalations, each of 7 days’ duration, after which dose escalation occurred as follows: FF (25, 100, 200, 400 and 800 µg/day), FP (50, 200, 500, 1000 and 2000 µg/day), BUD (100, 400, 800, 1600 and 3200 µg/day) or placebo on each day. All FF doses, FP 50 μg and BUD 100 μg doses were administered once daily in the evening; all other FP and BUD doses were administered twice daily (morning and evening, 12 h apart) to reflect the normal once-daily and twice-daily posology for FF, FP and BUD. Pre-dose samples were taken from each subject at the beginning of each treatment period and post-dose samples were taken from each subject at the end of each 7-day dose escalation cycle, on the morning of day 8, 12 h after the day 7 evening dose (Fig. 1). Participants abstained from eating/drinking (except water) overnight for ≥ 8 h prior to plasma sample collection. Samples were collected in ethylenediaminetetraacetic acid-containing tubes and immediately aliquoted into chilled polypropylene tubes and flash frozen prior to storage at approximately −80°C.

The study was approved by local ethics committees: Ethikkommission des Landes Berlin, Berlin, Germany (reference number 17/0432-EK 10), and North West – Greater Manchester South, NHS Health Research Authority, Manchester, UK (reference number 16/NW/0781).

### Subjects

The study population was previously described (16). Subjects were 18–65 years of age with ≥ 6 months’ history of mild bronchial asthma. At screening and at pre-dose baseline assessment, subjects had to demonstrate pre-bronchodilator forced expiratory volume in 1 s of ≥ 65% predicted. Short-acting beta-agonist use was allowed for ≥ 12 weeks prior to screening. Subjects who received a prescription of low-dose inhaled GCs could be included following 4-week washout without asthma control deterioration. Subjects who were light smokers (≤ 20 cigarettes/week) were included.

### Metabolomics

Plasma samples were analysed for changes to the global metabolome by Metabolon, Inc. (Morrisville, NC, USA). Analysis was conducted using an ultrahigh-performance liquid chromatography-tandem mass spectroscopy platform. Samples were analysed equally on various liquid chromatography-mass spectrometry (LC/MS) positive, negative and polar methods. Proprietary software matched ions to an in-house library of standards for metabolite identification and quantification. See Supplementary material (see section on supplementary materials given at the end of this article) for more details.

### Plasma cortisol

Determination of 24-h plasma cortisol has been described previously (16). Serial plasma samples were collected over the entire 24-h interval between post meridian (PM) on day 6 and PM on day 7 for each dose-escalation phase. These samples were analysed using LC/MS at Covance Bioanalytical Services (Indianapolis, IN, USA).

### Statistical analysis

Statistical analysis was by repeated-measures ANOVA, cross-over model, random forest and principal component analysis using natural log-transformed data and was performed in ArrayStudio v7.2 (R (v3.63) random Forest package or JMP were used for non-standard analyses). For all analyses, missing values were imputed with the observed overall minimum for that compound. Q-values (measuring false discovery rate) were calculated to account for false positives. Results are reported with adjusted *P*-values.

## Results

Baseline demographics were described previously; mean age was 37.9 years, 41 subjects (76%) were male, 39 subjects (72%) were White, and 19 subjects (35%) were current or former smokers (16). Mean forced expiratory volume in 1 s % predicted (s.d.) was 85.5 (12.9) (16). Overall, 54 subjects were included, with 18–21 subjects providing plasma samples for each inhaled GC at each dose level. Across all doses, few of the quantifiable metabolites (*n*  = 1971) had significant changes vs placebo (*P* < 0.05). For FF, FP and BUD, 1.34% (0.42% increased/0.92% decreased), 1.95% (0.41% increased/1.54% decreased) and 2.05% (0.60% increased/1.45% decreased) of the metabolites were significantly altered relative to placebo, respectively. The common therapeutic doses of inhaled GCs induced fewer significant changes: FF 100–200 µg/day, 0.96% (0.36% increased/0.61% decreased), FP 200–1000 µg/day, 1.66% (0.44% increased/1.22% decreased) and BUD 400–1600 µg/day, 1.45% (0.56% increased/0.90% decreased)). The highest doses and those exceeding approved therapeutic doses (FF ≥ 400 µg/day, FP ≥ 1000 µg/day and BUD ≥ 1600 µg/day) significantly affected several pathways: FF 1.78% (0.41% increased/1.37% decreased), FP 2.79% (0.51% increased/2.28% decreased), BUD 2.74% (0.23% increased, 2.51% decreased), as described below.

### Effects on adrenal steroid production

**Figure 2:**
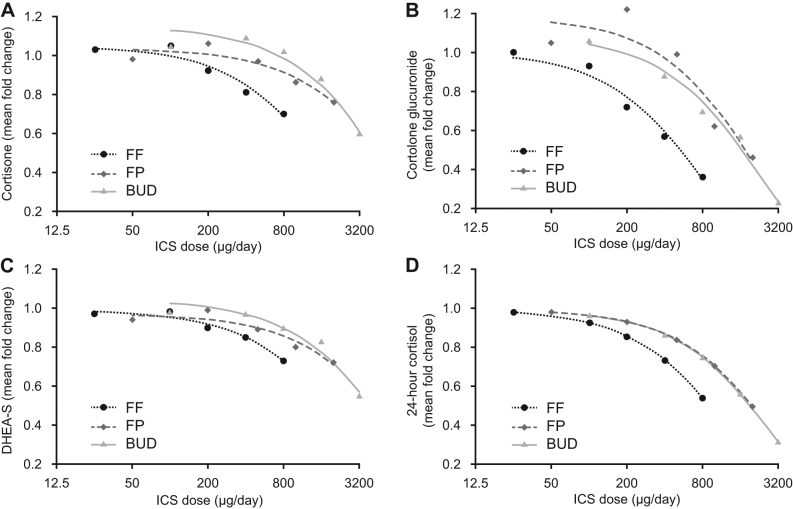
Relationship between dose and adrenal steroids measured via metabolomics (A) cortisone, (B) cortolone glucuronide and (C) DHEA-S all based on 12-h post-dose plasma sample on day 8 and (D*) 0–24-h-weighted mean plasma cortisol based on 24-h serial plasma samples taken between pre-PM dose on day 6 and pre-PM dose on day 7. *Image developed from data previously published in Daley-Yates *et al.* (2020) (16). BUD, budesonide; FF, fluticasone furoate; FP, fluticasone propionate; ICS, inhaled corticosteroid; PM, post meridian.

ln this analysis, several metabolites related to adrenal steroids were significantly reduced by inhaled GCs in a dose-dependent manner compared with placebo (Table 1). At high/supratherapeutic doses, all inhaled GCs reduced metabolites in GC pathways. Cortisone was significantly reduced (*P* ≤ 0.05) at FF 800 µg, BUD 3200 µg and FP 1000 and 2000 µg (mean fold change = 0.7 (i.e. 30% reduction), 0.6, 0.86 and 0.76, respectively) (Fig. 2A). Cortolone glucuronide levels exhibited a dose–response relationship for each molecule (Fig. 2B): it was significantly reduced at FF 200–800 µg, FP 1000–2000 µg and BUD 800–3200 µg; at the highest doses, the mean fold changes were 0.36, 0.46 and 0.23, respectively. Based on this single morning plasma sample collection, cortisol was significantly reduced vs placebo (*P* ≤ 0.05) at the highest dose of BUD (3200 µg), with a mean fold change of 0.8 (Table 1). However, the changes seen with the other inhaled GC doses were not statistically significant.

**Table 1:** Effects on adrenal steroid suppression. Biochemicals within the metabolomics data are fully annotated and identifications are qualified by Tier 1 standards according to the Metabolomics Society Initiative.

Pathways/biochemical	Fluticasone furoate (µg/day)					Fluticasone propionate (µg/day)					Budesonide (µg/day)				
	25	100	200	400	800	50	200	500	1000	2000	100	400	800	1600	3200
Glucocorticoid															
Cortisol	1.14	1.19↑	1.05	0.96	0.90	1.16	1.10	1.14	1.05	0.96	1.31↑	**1.23↑↑**	1.22	1.11	**0.80↓↓**
Cortisone	1.03	1.05	0.92↓	0.81↓	**0.70↓↓**	0.98	1.06	0.97	**0.86↓↓**	**0.76↓↓**	1.05	1.09	1.02	0.88↓	**0.60↓↓**
Cortolone glucuronide	1.00	0.93	**0.72↓↓**	**0.57↓↓**	**0.36↓↓**	1.05	1.22	0.99	**0.62↓↓**	**0.46↓↓**	1.06	0.88↓	**0.70↓↓**	**0.57↓↓**	**0.23↓↓**
Androgenic steroids															
DHEA-S	0.97	0.98	**0.90↓↓**	**0.85↓↓**	**0.73↓↓**	0.94	0.99	**0.89↓↓**	**0.80↓↓**	**0.72↓↓**	0.98	0.97	**0.90↓↓**	**0.83↓↓**	**0.55↓↓**
Androsterone glucuronide	1.06	1.00	1.04	0.97	**0.79↓↓**	0.96	0.95	**0.89↓↓**	**0.73↓↓**	**0.72↓↓**	1.05	1.11↑	0.99	0.91↓	**0.64↓↓**
Epiandrosterone sulfate	0.98	0.96	0.99	0.96	0.99	**0.89↓↓**	1.01	0.95	**0.89↓↓**	**0.85↓↓**	0.94	0.95	1.00	**0.89↓↓**	**0.78↓↓**
Androsterone sulfate	1.03	0.99	1.00	0.97	0.99	0.94↓	1.01	0.97	0.90↓	**0.84↓↓**	0.98	0.99	0.98	**0.92↓↓**	**0.77↓↓**
Androstenediol (3beta, 17beta) disulfate	1.03	1.01	0.97	0.97	**0.87↓↓**	0.95	1.01	0.96	0.94	**0.86↓↓**	0.99	1.02	1.01	**0.92↓↓**	**0.71↓↓**
Pregnenolone steroids															
21-Hydroxypregnenolone disulfate	1.06	1.09	0.93	0.87↓	**0.71↓↓**	1.06	1.06	0.98	0.94	**0.79↓↓**	1.19↑	1.11↑	1.02	**0.84↓↓**	**0.50↓↓**
Pregnenediol sulfate*	0.94↓	1.02	**0.89↓↓**	**0.81↓↓**	**0.69↓↓**	0.97	0.99	0.96	**0.83↓↓**	**0.74↓↓**	1.17	1.08	1.02	**0.87↓↓**	**0.54↓↓**
Pregnenediol disulfate*	1.04	1.07	**0.92↓↓**	0.90↓	**0.84↓↓**	0.97	0.98	**0.88↓↓**	**0.85↓↓**	**0.77↓↓**	1.07	1.03	0.98	**0.89↓↓**	**0.67↓↓**
Pregnenetriol sulfate	0.93↓	1.01	**0.84↓↓**	**0.79↓↓**	**0.69↓↓**	0.99	1.08	0.97	**0.88↓↓**	**0.77↓↓**	1.08	1.01	0.95	**0.85↓↓**	**0.53↓↓**
Pregnenetriol disulfate	1.01	1.05	**0.89↓↓**	0.86↓	**0.80↓↓**	0.95	0.99	**0.89↓↓**	**0.86↓↓**	**0.70↓↓**	1.00	0.97	0.94↓	**0.84↓↓**	**0.55↓↓**

↑Increase 0.05 < *P* < 0.10; ↓Decrease 0.05 *<* *P* < 0.10; **↑↑**Significant (*P* ≤ 0.05) increase; **↓↓**Significant (*P* ≤ 0.05) decrease. *Compounds that have not been officially confirmed based on a standard, despite confidence in their identities, are designated as Tier 2.

**Figure 3:**
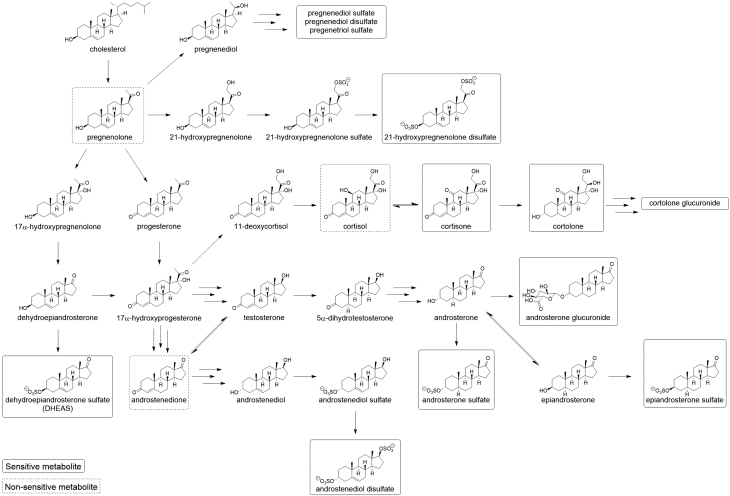
Steroid molecule pathways. All structures available from PubChem database at https://pubchem.ncbi.nlm.nih.gov/.

Among components of androgenic steroid pathways, a dose–response relationship was seen for DHEA-S, which was significantly reduced at high/supratherapeutic doses for all three inhaled GCs, but not at lower doses (Fig. 2C and Table 1). Changes were observed for pregnenolone steroids (Fig. 3), most notably pregnenediol sulfate, pregnenediol disulfate, pregnenetriol sulfate and pregnenetriol disulfate, which were all significantly reduced (*P* < 0.05) at higher doses for all three inhaled GCs (Table 1).

### Effects on collagen-associated amino acids and branched-chain amino acids

There were small but statistically significant (*P* ≤ 0.05) increases in plasma amino acid levels (serine, alanine, asparagine, phenylalanine and methionine), particularly with FF (Table 2). Serine and asparagine (mean fold changes = 1.07–1.11 and 1.09–1.14, respectively) were significantly increased at all FF doses. Asparagine significantly increased with BUD 400 and 800 µg, but not other doses. All doses of FF significantly increased phenylalanine and methionine (mean fold changes = 1.08–1.10 and 1.17–1.24, respectively), except for 100 µg. Phenylalanine was also significantly increased at certain doses of BUD (100, 400 and 800 µg; mean fold change = 1.08–1.11), while methionine was significantly increased at certain doses of FP (200 and 1000 µg; mean fold change = 1.13–1.36). Alanine was significantly increased only with FF 25 µg (*P* = 0.013).

**Table 2:** Effects on collagen-associated amino acids and branched-chain amino acids: proteolysis, bone and connective tissue metabolism.

Pathways/biochemical	Fluticasone furoate (µg/day)					Fluticasone propionate (µg/day)					Budesonide (µg/day)				
	25	100	200	400	800	50	200	500	1000	2000	100	400	800	1600	3200
Amino acids															
Serine	**1.10↑↑**	**1.08↑↑**	**1.07↑↑**	**1.11↑↑**	**1.10↑↑**	1.06	1.01	1.02	1.07	1.08	1.02	1.06	1.07	1.03	1.04
Alanine	**1.09↑↑**	1.04	1.09	1.09	1.05	1.06	1.05	0.96	1.00	1.07	1.09	1.13↑	1.09	1.06	1.05
Asparagine	**1.11↑↑**	**1.09↑↑**	**1.14↑↑**	**1.14↑↑**	**1.14↑↑**	1.02	1.00	0.97	1.03	1.04	1.07	**1.1↑↑**	**1.11↑↑**	1.08	1.06↑
Phenylalanine	**1.08↑↑**	1.04	**1.1↑↑**	**1.08↑↑**	**1.08↑↑**	1.02	1.04	0.98	1.04	1.08	**1.08↑↑**	**1.09↑↑**	**1.11↑↑**	1.08↑	1.07↑
Methionine	**1.17↑↑**	1.02	**1.24↑↑**	**1.20↑↑**	**1.15↑↑**	1.12	**1.13↑↑**	1.05	**1.36↑↑**	1.23↑	1.08	1.13	1.10	1.08	**1.13↑↑**
Proline	1.04	1.06	1.05	1.01	1.03	1.08	1.09	1.02	1.09	1.08	1.06	1.06	1.08	1.02	1.00
3-Methylhistidine	3.62	4.57	5.07	4.59	4.21	2.31	2.89	2.66	2.86	2.79	4.68	2.80	4.03	3.49	2.91
*Trans*-4-hydroxyproline	0.91↓	**0.83↓↓**	**0.85↓↓**	**0.75↓↓**	0.91↓	**0.88↓↓**	**0.89↓↓**	0.92↓	**0.87↓↓**	0.89↓	1.02	1.13	0.94	0.89↓	**0.84↓↓**
Branched-chain amino acids															
Leucine	**1.08↑↑**	1.07	**1.15↑↑**	**1.08↑↑**	**1.10↑↑**	1.06	**1.08↑↑**	1.00	**1.07↑↑**	1.10	**1.08↑↑**	**1.11↑↑**	**1.14↑↑**	1.09↑	1.02
*N*-Acetylleucine	1.05	1.02	1.18	1.18↑	1.08	1.13	**1.17↑↑**	1.11	1.17↑	**1.31↑↑**	1.04	1.14	1.15↑	1.12	1.04
4-Methyl-2-oxopentanoate	1.07	0.92	0.97	1.05	0.97	1.04	1.07	1.01	1.04	1.01	**1.17↑↑**	**1.12↑↑**	1.13	1.16	**1.22↑↑**
Alpha-hydroxyisocaproate	**1.18↑↑**	1.09	1.15	1.16↑	1.08	1.01	1.09	1.07	0.99	0.94	**1.35↑↑**	**1.26↑↑**	**1.38↑↑**	**1.37↑↑**	**1.21↑↑**
2-Ketocaprylate	**0.74↓↓**	**0.79↓↓**	**0.72↓↓**	1.02	1.14	**0.82↓↓**	**0.77↓↓**	0.93	**0.72↓↓**	**0.78↓↓**	**0.86↓↓**	**0.80↓↓**	**0.66↓↓**	0.95↓	1.04↑
Isovalerate (i5:0)	1.42	1.22	1.43	1.18	**1.75↑↑**	1.10	0.97	1.20	1.03	1.15	1.18	1.22	1.18	1.23	1.21
Isovalerylcarnitine (C5)	1.23↑	1.15	1.20	1.22	**1.53↑↑**	0.97	1.04	1.10	1.07	1.14	1.02	1.09	1.25	1.14	1.20
Beta-hydroxyisovalerate	0.93	0.96	1.07	1.09	1.14	**0.90↓↓**	1.03	0.99	1.04	1.08	1.15	1.26↑	**1.3↑↑**	**1.28↑↑**	**1.34↑↑**
Isoleucine	1.02	1.01	**1.10↑↑**	**1.07↑↑**	**1.07↑↑**	1.08	**1.10↑↑**	1.03	**1.10↑↑**	1.16↑	1.02	1.08↑	**1.10↑↑**	1.06	1.01
3-Methyl-2-oxovalerate	1.03	0.91	0.97	1.06	1.00	1.07	1.09	1.05	1.08	1.08	1.10	1.11↑	1.09	1.16	**1.17↑↑**
2-Methylbutyrylcarnitine (C5)	0.98	**0.92↓↓**	0.98	1.06	1.08	**0.85↓↓**	0.94	0.96	**0.89↓↓**	0.92↓	0.97	1.03	1.00	0.98	0.96
2-Methylbutyrylglycine	1.54	1.77↑	1.39	**2.14↑↑**	1.97	1.56	1.46	1.65	2.13	1.28	1.20	1.23	1.27↑	1.32↑	1.18
Tiglylcarnitine (C5:1-DC)	1.06	1.17	1.04	1.08	1.12	0.92↓	0.99	1.03	0.96	0.94	1.07	1.09	1.16	1.04	1.15
3-Hydroxy-2-ethylpropionate	0.98	0.97	**0.88↓↓**	0.91↓	1.00	**0.89↓↓**	0.95	0.94	**0.87↓↓**	**0.89↓↓**	1.06	1.09	1.09	1.04	1.06
Ethylmalonate	1.00	0.98	1.00	0.97	1.02	**0.85↓↓**	0.93↓	**0.91↓↓**	**0.9↓↓**	0.97	1.03	0.96	1.08	1.00	0.94
Valine	**1.08↑↑**	1.07	**1.12↑↑**	**1.09↑↑**	**1.11↑↑**	1.00	1.03	0.99	1.01	1.03	1.10	1.09	**1.16↑↑**	1.10	1.04
*N*-Acetylvaline	1.06	1.05	1.06	1.05	1.09	0.94↓	0.96	0.94↓	0.97	1.01	1.05	**1.14↑↑**	1.11↑	1.12↑	1.09
Alpha - hydroxyisovalerate	**0.91↓↓**	0.96	0.93↓	0.96	0.93↓	**0.88↓↓**	0.97	0.93↓	**0.91↓↓**	0.92↓	1.04	1.05	1.15↑	1.13	1.01
Isobutyrylglycine	1.48↑	1.50	**1.61↑↑**	2.02	1.65	1.01	1.56	1.68	1.20	1.34	1.10	0.98	1.10	1.01	1.10

↑Increase 0.05 < *P* < 0.10; ↓Decrease 0.05 < *P* < 0.10; **↑↑**Significant (*P* ≤ 0.05) increase; **↓↓**Significant (*P* ≤ 0.05) decrease.

There were statistically significant (*P* ≤ 0.05) decreases in *trans*-4-hydroxyproline (a collagen-associated amino acid) across all treatment groups, but there were no significant changes in 3-methylhistidine (a marker of muscle turnover) in any treatment group. Effects of inhaled GCs on branched-chain amino acids (BCAAs) were observed: leucine, isoleucine and valine were significantly (*P* < 0.05) increased in plasma at therapeutic and supratherapeutic doses. Levels of some BCAA catabolic intermediates (1-carboxyethyl-leucine, 2-ketocaprylate, 3-methyl-glutaconate, 1-carboxyethyl-isoleucine, 2-methyl-butyrylcarnitine (C5), 3-hydroxy-2-ethyl-propionate, ethyl-malonate, methyl-succinate, alpha-hydroxy-isovalerate) were significantly decreased by inhaled GCs.

### Effects on the immune system and inflammation

Several anti-inflammatory mediators and precursors derived from polyunsaturated fatty acid metabolites were significantly decreased by inhaled GCs. In subjects treated with FP and BUD, mean fold changes in eicosapentaenoate were 0.85–0.86 (for 200 and 1000 µg doses) and 0.83 (for 1600 µg dose only), respectively (*P* ≤ 0.05). Significant decreases were observed in docosapentaenoate, docosahexaenoate, dihomo-linoleate and arachidonate in these subjects (Table 3). No significant changes were observed in these metabolites with FF treatment.

**Table 3:** Effects on the immune system and inflammation: anti-inflammatory effects, modulation of anti-inflammatory mediators and precursors.

Inflammatory mediators, PUFA metabolites	Fluticasone furoate (µg/day)					Fluticasone propionate (µg/day)					Budesonide (µg/day)				
	25	100	200	400	800	50	200	500	1000	2000	100	400	800	1600	3200
Eicosapentaenoate (EPA; 20:5n3)	0.97	1.25	0.97	1.00	1.04	0.97	**0.85↓↓**	0.89↓	**0.86↓↓**	0.91↓	1.20	0.95	1.02	**0.83↓↓**	1.02
Docosapentaenoate (n3 DPA; 22:5n3)	1.04	1.33	1.10	1.12	1.32	0.92↓	**0.75↓↓**	0.92↓	**0.91↓↓**	**0.88↓↓**	1.43	1.09	0.92	**0.81↓↓**	1.08
Docosahexaenoate (DHA; 22:6n3)	0.95	1.24	1.06	1.04	1.07	0.93↓	**0.78↓↓**	0.90↓	**0.86↓↓**	**0.81↓↓**	1.41	1.05	0.91↓	**0.79↓↓**	1.03
Dihomo-linoleate (20:2n6)	1.09	1.40	1.10	1.21	1.41	**0.89↓↓**	**0.70↓↓**	0.95	0.99↓	0.95	1.27	0.96	0.89↓	**0.81↓↓**	0.99
Arachidonate (20:4n6)	1.04	1.36	1.14	1.18	1.34	0.97	**0.85↓↓**	1.02	1.03	0.99	1.28	1.05	1.03	**0.87↓↓**	1.12
Sphingosine 1-phosphate	1.06	0.97	1.01	1.05	1.00	0.98	1.02	1.03	1.02	1.02	1.04	1.01	1.18	1.05	1.04

↑Increase 0.05 < *P* < 0.10; ↓Decrease 0.05 < *P* < 0.10; **↑↑**Significant (*P* ≤ 0.05) increase; **↓↓**Significant (*P* ≤ 0.05) decrease.

PUFA, polyunsaturated fatty acid.

### Effects on energy metabolism

Overall, the results provided little evidence of energy pathway disruption (increased gluconeogenesis or reduced glucose utilisation). Although BUD appeared to cause small increases in plasma glucose, there was no dose–response (Table 4). However, there was some indication of a shift in energy utilisation, with an increase in glycolysis – evidenced by a significant increase in 3-phosphoglycerate and a significant decrease in intermediates of the TCA cycle (citrate and aconitate (*cis* or *trans*)). Mean fold changes in 3-phosphoglycerate were 1.55–2.68 and 2.24 with FP (1000 and 2000 µg doses) and BUD (800 µg dose only), respectively. No significant changes were observed in 3-phosphoglycerate with FF.

**Table 4:** Effects on energy metabolism: glycolysis, TCA and urea cycles, gluconeogenesis, glucose utilisation, catabolism and lipolysis

Biochemical	Fluticasone furoate (µg/day)					Fluticasone propionate (µg/day)					Budesonide (µg/day)				
	25	100	200	400	800	50	200	500	1000	2000	100	400	800	1600	3200
Glucose	1.02	1.02	1.02	0.98	1.02	1.01	1.03	1.01	1.01	1.04	**1.10↑↑**	**1.05↑↑**	1.07↑	1.00	0.99
3-Phosphoglycerate	1.90	1.17	1.47	1.62	1.40	1.54	1.48	1.54	**1.55↑↑**	**2.68↑↑**	1.09	1.10	**2.24↑↑**	1.57	1.56
Lactate	1.23	1.07	1.11	1.32	1.13	1.08	1.01	1.09	1.13	1.17	1.19	1.18	1.11	1.16	1.06
Mannitol/Sorbitol	1.21	1.15	**0.81↓↓**	1.07	0.91↓	1.24	1.09	0.98↓	0.86↓	1.07	**0.81↓↓**	**0.86↓↓**	0.87↓	0.89↓	**0.73↓↓**
Mannose	1.11	1.15↑	1.14	1.09	1.23	0.94	1.02	1.06	1.00	1.02	1.10	1.07	**1.14↑↑**	1.09	1.05
Citrate	0.96↓	0.94↓	0.96	**0.92↓↓**	**0.85↓↓**	**0.93↓↓**	**0.93↓↓**	**0.90↓↓**	**0.90↓↓**	0.93↓	1.07↑	1.00	1.03	0.98	**0.90↓↓**
Aconitate (*cis* or *trans*)	0.91↓	**0.87↓↓**	**0.88↓↓**	**0.88↓↓**	**0.82↓↓**	**0.89↓↓**	**0.87↓↓**	0.91↓	**0.92↓↓**	0.92	1.01	0.94	1.06	0.95	**0.90↓↓**
3-Hydroxybutyrate (BHBA)	1.15	1.68	1.08	1.54	2.23	1.25	**0.82↓↓**	1.75	1.51	1.81	1.50	1.03	1.65	1.32	1.29
Glycerol	0.96	1.00	0.88↓	0.95	0.96	1.12	1.01	**0.80↓↓**	**0.78↓↓**	0.93	0.99	**0.80↓↓**	1.04	**0.87↓↓**	**0.85↓↓**

↑Increase 0.05 < *P* < 0.10; ↓Decrease 0.05 < *P* < 0.10; **↑↑**Significant (*P* ≤ 0.05) increase; **↓↓**Significant (*P* ≤ 0.05) decrease.

TCA, tricarboxylic acid.

**Figure 4:**
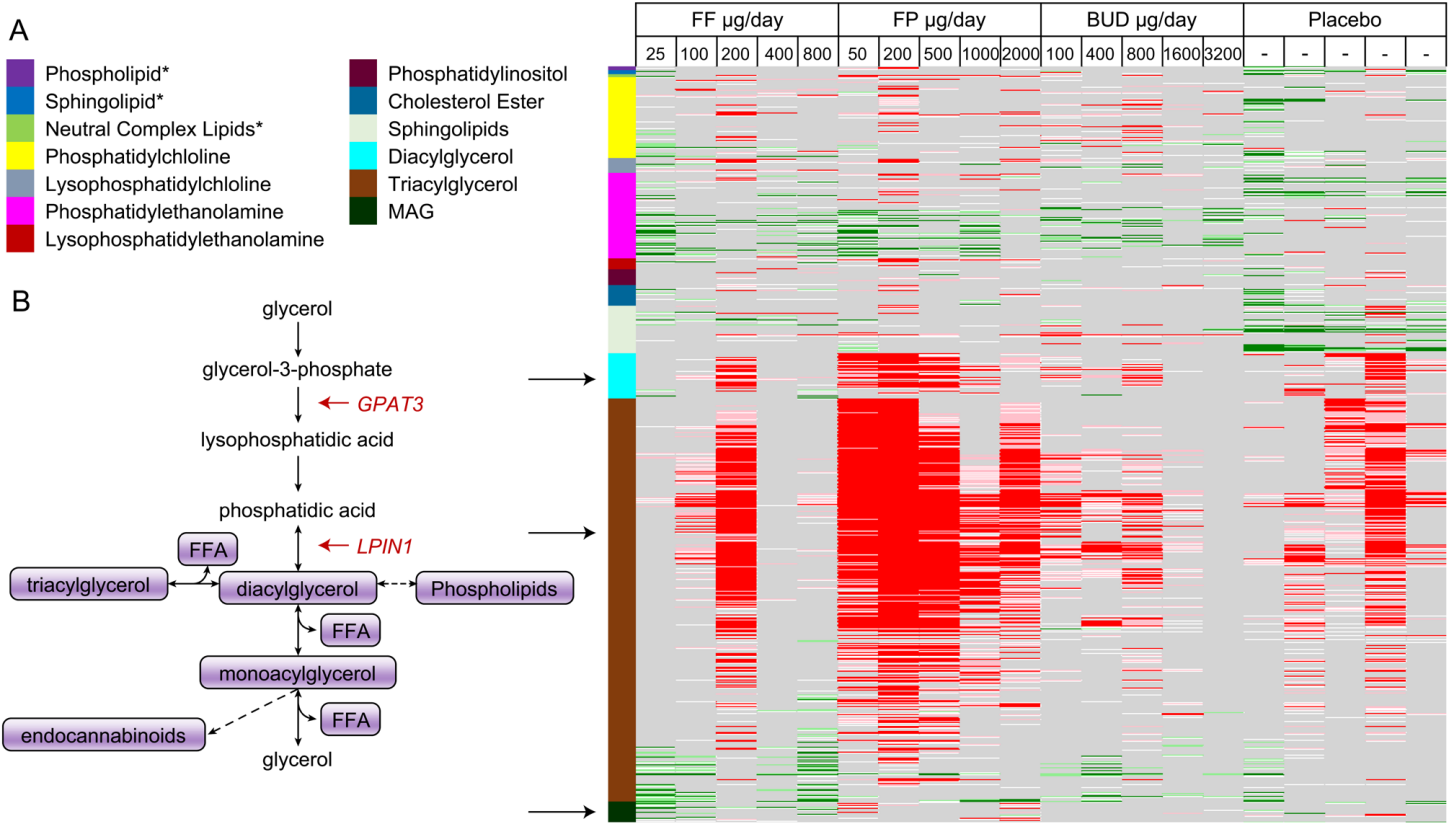
Intra-individual comparison of complex lipids. (A) Arrows point towards increased TAG and DAG species and decreased MAG species. Red boxes in the heat map represent significantly increased biochemicals (*P* ≤ 0.05). Green boxes in the heat map represent significantly decreased biochemicals (*P* ≤ 0.05). Pink and light green boxes represent biochemicals which are trending towards a significant increase or decrease, respectively (0.05 < *P* < 0.10). (B) TAG synthesis and hydrolysis pathways. *The sum of molecules in each class. BUD, budesonide; DAG, diacylglycerol; FF, fluticasone furoate; FP, fluticasone propionate; FFA, free fatty acids; GPAT3, glycerol-3-phosphate acyltransferase 3; LPIN1, Lipin1; MAG, monoacylglycerol; TAG, triacylglycerol.

There was some evidence of reduced fatty acid β-oxidation (Fig. 4); all treatments (including placebo) were associated with increased triacylglycerol (TAG) plasma levels. However, FP showed the highest increase in TAG and diacylglycerol (DAG) levels and significant decreases in free fatty acids (FFAs).

### Effects on neurotransmitters

Subjects who received escalating doses of BUD exhibited significantly lower levels of 3-methoxytyramine sulfate (a dopamine metabolite) and dopamine 3-*O*-sulfate (the predominant regioisomer of dopamine in humans) (Table 5) (17, 18). The mean fold changes in 3-methoxytyramine sulfate were 0.76 (for 400 µg dose; *P* = 0.009), 0.74 (800 µg; *P* = 0.006) and 0.72 (3200 µg; *P* < 0.001). Mean fold changes in dopamine 3-*O*-sulfate were 0.81 (for 800 µg dose; *P* = 0.033), 0.82 (1600 µg; *P* = 0.047) and 0.72 (3200 µg; *P* = 0.014). No dose-related effects were seen with FF or FP for these metabolites.

**Table 5:** Effects on neurotransmitters, catecholeamines, amino acid precursors and metabolites: immune regulation, psychological and behavioural effects.

Biochemical	Fluticasone furoate (µg/day)					Fluticasone propionate (µg/day)					Budesonide (µg/day)				
	25	100	200	400	800	50	200	500	1000	2000	100	400	800	1600	3200
Tyrosine	1.03	0.98	1.08↑	1.05	1.05	1.06	1.07	0.98	0.97	1.08	1.06	1.08↑	1.06	1.06	1.03
3-(4-Hydroxyphenyl)lactate	1.07	1.00	1.04	1.02	1.02	0.97	1.06	1.00	0.97	1.04	**1.09↑↑**	**1.10↑↑**	1.12	1.14↑	1.08↑
3-Methoxytyramine sulfate	1.05	0.90↓	0.96	0.97	1.02	1.03	1.03	1.20	0.97	**0.88↓↓**	0.91	**0.76↓↓**	**0.74↓↓**	0.87↓	**0.72↓↓**
Homovanillate (HVA)	0.99	1.03	1.11	1.00	1.13	1.02	0.99	0.90↓	**0.87↓↓**	0.96	1.23	1.09	1.03	1.04	1.03
Dopamine 4-sulfate	1.08	1.20	1.17	1.71	1.35	1.29	1.57	2.44	1.42	1.88	1.62	1.21	**1.10↑↑**	1.10	0.83↓
Dopamine 3-O-slufate	0.98	0.89↓	0.93	1.43	0.99	1.19	1.17	1.36	1.08	1.28	1.11	0.90↓	**0.81↓↓**	**0.82↓↓**	**0.72↓↓**
Tryptophan	1.01	0.99	1.07	1.06↑	1.07	1.01	1.07↑	1.01	1.08	**1.13↑↑**	1.05	**1.12↑↑**	**1.15↑↑**	1.12↑	1.09
Kynurenine	1.10	1.04	1.02	1.01	0.97	0.93↓	1.02	0.99	0.99	0.96	1.06	**1.11↑↑**	1.12	1.01	0.94↓
Xanthurenate	1.17	1.14	1.19	1.23	1.17	0.91↓	0.90↓	0.90↓	**0.85↓↓**	**0.87↓↓**	1.44	1.28	1.17	1.17	0.93
8-Methoxykynurenate	1.14	1.14	1.08	1.06	1.01	**0.87↓↓**	0.90↓	**0.87↓↓**	**0.77↓↓**	**0.84↓↓**	1.51	1.22	1.22	1.11	1.02
Serotonin	3.16	1.91	3.15	4.54	7.77	1.72	5.80	4.38	2.01	**4.83↑↑**	1.96	2.34	4.53	2.70	6.82↑
Indolelactate	0.95	0.93↓	0.98	1.00	0.97	**0.88↓↓**	0.94↓	**0.92↓↓**	**0.89↓↓**	0.95	1.03	1.04	1.11	1.10	0.98
Indoleacetate	1.03	0.94	1.14	1.02	1.07	0.91↓	1.02	0.92	0.94	1.04	1.02	1.21	1.08	1.07	1.00
3-Indoxyl sulfate	0.98	1.19	1.04	0.98	1.09	1.08	1.16	1.10	0.99	1.16	1.11	1.17	1.16	0.99	1.03

↑Increase 0.05 < *P* < 0.10; ↓Decrease 0.05 < *P* <0.10; **↑↑**Significant (*P* ≤ 0.05) increase; **↓↓**Significant (*P* ≤ 0.05) decrease.

### Effects on microbiota-derived metabolites

There were notable qualitative changes in secondary bile acid metabolites (Table 6). These were most readily observed for BUD and FF. Furthermore, the aromatic amino acid metabolites phenyllactate and 3-(4-hydroxyphenyl)lactate, which are generated by the intestinal microbiota, significantly increased with BUD in a dose-dependent manner (Table 6).

**Table 6:** Effects on secondary bile acids. Biochemicals within the metabolomics data are fully annotated and identifications are qualified by Tier 1 standards according to the Metabolomics Society Initiative.

Biochemical	Fluticasone furoate (µg/day)					Fluticasone propionate (µg/day)					Budesonide (µg/day)				
	25	100	200	400	800	50	200	500	1000	2000	100	400	800	1600	3200
Secondary bile acids															
Deoxycholate	1.41	1.16	1.09	1.28	1.74	1.73	1.71	1.75	2.47	1.47	1.41	0.98	1.71	1.10	1.01
Deoxycholic acid (12 or 24)-sulfate*	1.64	1.49	1.18	1.63	**1.75↑↑**	1.49	1.18	1.17	1.28	1.65	1.45↑	**1.64↑↑**	**2.05↑↑**	1.37	1.45
Deoxycholic acid glucuronide	**0.89↓↓**	1.09	0.87↓	0.9↓	1.00	1.25	1.21	1.15	1.13	1.16	0.91↓	0.90↓	1.12	0.92↓	**0.85↓↓**
Glycodeoxycholate	**3.57↑↑**	4.57↑	3.43	3.46	3.43	**3.3↑↑**	2.17	2.27	2.34↑	1.63	3.05	1.77	3.13	2.77	1.25
Taurodeoxycholate	**7.82↑↑**	**2.29↑↑**	1.93	3.76	4.21↑	4.10	3.18	4.67↑	6.98↑	2.92	6.50↑	2.52	7.70	5.35	1.54
Taurodeoxycholic acid 3-sulfate	2.24↑	1.84	1.75↑	**2.13↑↑**	**2.30↑↑**	1.35	1.16	1.67	2.16	2.36	2.46↑	1.85	2.28↑	2.43	2.73
Lithocholate sulfate	1.54	1.63	1.63	1.77	**2.45↑↑**	1.61	1.21	1.12	1.41	1.53	**2.12↑↑**	**2.37↑↑**	**2.35↑↑**	1.99	1.77
Lithocholic acid sulfate	1.05	1.72	1.27	1.03	2.18	2.47	1.76	1.73	2.24	2.34	1.97	1.70	**3.01↑↑**	1.68	2.03
Glycolithocholate	4.89↑	**4.39↑↑**	2.29↑	**2.98↑↑**	**3.59↑↑**	2.04	2.55	3.28	**2.36↑↑**	**3.37↑↑**	**4.51↑↑**	**2.84↑↑**	**2.92↑↑**	**2.88↑↑**	2.21
Glycolithocholate sulfate*	**1.62↑↑**	1.43	**1.73↑↑**	1.93	**2.19↑↑**	1.82↑	1.36	1.60	1.57	**2.03↑↑**	**2.20↑↑**	**2.15↑↑**	**2.68↑↑**	**2.07↑↑**	1.82↑
Taurolithocholate 3-sulfate	**1.93↑↑**	1.55	1.95↑	1.95	**2.17↑↑**	1.64	1.21	1.53	1.82	2.01↑	**3.71↑↑**	**3.7↑↑**	**4.64↑↑**	**2.85↑↑**	3.08↑
Ursodeoxycholate	1.52	3.39	**0.95↓↓**	1.35	1.50	4.85	**7.94↑↑**	**4.53↑↑**	5.94	5.72	2.60	2.70	8.71	1.01	1.94
Isoursodeoxycholate	1.40	2.39↑	1.36	1.74	1.31	1.25	1.38	1.31	1.14	1.34	1.27	1.25↑	1.23	1.14	1.25
Glycoursodeoxycholate	3.18↑	**3.36↑↑**	1.76	2.17	2.77	**1.87↑↑**	1.58↑	**2.10↑↑**	1.72↑	1.42	1.49	1.27	1.48	1.56	1.05
Glycoursodeoxycholic acid sulfate	0.90	1.59	0.99	1.22	1.78	2.01	1.38	2.34	2.01	2.27	1.08	1.07	0.91↓	1.34	1.37
Hyocholate	**0.68↓↓**	0.91↓	**0.62↓↓**	**0.76↓↓**	0.98	**0.85↓↓**	0.92↓	0.93↓	1.10	1.34	**0.87↓↓**	**0.82↓↓**	1.04	0.89↓	1.04
Glycohyocholate	**2.13↑↑**	**2.02↑↑**	1.75	**2.63↑↑**	**3.17↑↑**	1.76	1.37	2.07	1.58	1.44	1.60	1.51	1.37	1.37	1.44
Glycocholenate sulfate*	1.01	1.09	1.02	1.08	**1.18↑↑**	1.09	1.12	1.08	1.09	**1.29↑↑**	**1.18↑↑**	**1.16↑↑**	**1.3↑↑**	**1.14↑↑**	1.16↑
Taurocholenate sulfate*	1.32↑	1.14	1.42	1.47↑	**1.77↑↑**	1.32	1.17	1.26	1.46	1.31	1.61↑	1.46	1.57	1.40	1.51
3b-Hydroxy-5-cholenoic acid	0.92	1.14	0.90↓	0.98	1.17	**0.89↓↓**	1.02	1.05	1.12	1.15	1.00	1.11	1.11	0.94	1.05
Glycodeoxycholate 3-sulfate	1.56	2.01↑	1.77	**2.23↑↑**	**2.21↑↑**	1.57↑	1.06	1.62	1.38	1.50	2.39↑	2.69	**3.67↑↑**	**3.18↑↑**	3.08↑
Taurochenodeoxycholic acid 3-sulfate	1.88	2.83	1.90	**2.51↑↑**	3.60	2.77	2.05	2.65	2.31	2.68	2.61↑	3.30	3.91	2.26	2.50
Aromatic amino acids															
3-(4-Hydroxyphenyl)lactate	1.07	1.00	1.04	1.02	1.02	0.97	1.06	1.00	0.97	1.04	**1.09↑↑**	**1.1↑↑**	1.12	1.14↑	1.08↑
Phenyllactate	1.03	1.00	1.01	1.03	1.07	0.97	1.00	0.96	0.97	0.99	**1.09↑↑**	1.05	**1.16↑↑**	**1.14↑↑**	**1.13↑↑**

↑Increase 0.05 < *P* < 0.10; ↓Decrease 0.05 < *P* < 0.10; **↑↑**Significant (*P* ≤ 0.05) increase; **↓↓**Significant (*P* ≤ 0.05) decrease. *Compounds that have not been officially confirmed based on a standard, despite confidence in their identities, are designated as Tier 2.

## Discussion

Widespread systemic metabolomic changes were seen with inhaled FF, FP and BUD; many of these occurred in a dose-dependent manner but the largest and statistically significant changes were seen predominately at high and supratherapeutic doses. These changes included dose-related reductions in adrenal steroids; increases in amino acids and BCAAs; decreases in anti-inflammatory mediators; some evidence of an energy utilisation shift (increased glycolysis); and a reduction in TCA intermediates. Subjects who received escalating doses of BUD had significantly lower levels of dopamine 3-*O*-sulfate and 3-methoxytyramine sulfate compared with placebo. Many observed changes were within ± 20%, whether identified as statistically significant or not; it is unlikely that these were biologically or clinically significant because of the absence of a dose–response coupled with a small effect size. Many of the pathways susceptible to GCs are highly regulated by physiological mechanisms to maintain homeostasis; therefore, large changes may not be seen until significant metabolic dysregulation occurs and based on our findings this is only likely at high or supratherapeutic doses of inhaled GCs.

Metabolomic analysis of a single plasma sample taken on the morning of day 8, 12 h after the last inhaled GCs dose, revealed decreases in adrenal steroid pathway metabolites. These changes were significant and dose-dependent at higher doses of inhaled GCs, although FF had the least effect on these pathways. Notably, at the lowest therapeutic dose of FF (100 µg/day), there were no statistically significant changes. Significant changes in cortisol, when assessed at the single timepoint of 12 h post-dose, were only seen with the 3200 µg/day of BUD, which showed a significant reduction (*P* ≤ 0.05) of 20%, whereas the glucuronide metabolites of cortisol and cortisone and the pregnenolone metabolite DHEA-S exhibited the largest dose-related reductions. In contrast, the previously published primary endpoint for this study of 24-h weighted mean plasma cortisol, based on serial blood sampling between the pre-PM dose day 6 and pre-PM dose day 7, showed well-defined dose–responses for each inhaled GCs (Fig. 2D). Doses of FF ≥ 400 µg, FP ≥ 1000 µg and BUD ≥ 800 µg exhibited significant reductions in plasma cortisol, with the mean and 95% CI indicating > 20% reductions with the greatest (mean, 95% CI) reduction seen with 3200 µg/day of BUD (68.4%, 63.7–72.4%) (16). The widespread reductions in adrenal steroids, including cortisol, cortisone/cortolone glucuronides and pregnenolone, indicate that all three inhaled GCs could potentially impact HPA axis function and that only measuring cortisol, particularly at a single timepoint, may not fully reflect this effect. The likely mechanism for this effect is the presence of circulating exogenous GCs disrupting biofeedback within the HPA axis, suppressing the synthesis of endogenous steroids, their precursors and metabolites. Measurements of other HPA axis components, such as adrenocorticotropic hormone and corticotrophin-releasing hormone, were not done as the focus of this work was metabolomics rather than proteomics but such measurements could be potentially informative and should be considered in future work. Therefore, the decrease in adrenal metabolites found in this study may indicate the potential for adrenal suppression to occur in subjects receiving high doses of inhaled GCs for extended periods as reported elsewhere (19, 20).

Whereas other metabolomic studies have focused on indices for prediction of the incidence and severity of asthma in adults and children (8, 9, 10, 11, 12, 13, 14, 15), here we studied the systemic effects of the most common asthma pharmacotherapy. Broadly, at low doses, the metabolic changes seen in adrenal steroid pathways were < 20% and not statistically significant, in agreement with data for 24-h plasma cortisol suppression data in the same subjects where FF 100 and 200 μg/day induced similar cortisol suppression (7.41 and 14.28%, respectively) compared to FP 200 and 500 μg/day (6.74 and 16.01%, respectively) and BUD 200 and 400 μg/day (7 and 13.4%, respectively) (16).

Increases were seen in plasma amino acids, but mean fold changes were mostly < 20%, with little evidence of dose–response. Plasma amino acids can indicate changes to extracellular matrix (ECM) composition. As Hoshino *et al.* showed, inhaled GC treatment can lead to ECM remodelling via the activation of collagen-degrading metalloproteases (21). Therefore, changes in the levels of collagen-associated amino acids and amino acid derivatives could be indicative of changes in ECM biology. It is possible that FF influences tissue structure/function most by modulating ECM and fibrin biology. Plasma amino acid levels were found to increase as the dose of inhaled GC increased, particularly for FF and BUD, possibly suggesting dose-related proteolysis. The site of proteolysis remains unknown; however, inhaled GCs differ in their lung retention (22), hence, with FF, proteolysis may occur to a greater extent in pulmonary tissue as it has higher lung retention (22). This may result in relatively more systemic proteolysis with BUD and translate to a greater risk of skin thinning, which is a recognised side-effect of inhaled GCs (23).

Inhaled GCs caused plasma enrichment with BCAAs at the highest doses studied, while the levels of BCAA catabolic intermediates decreased. As BCAAs are essential for the maintenance of globular protein structures, changes could indicate protein degradation (24). Free BCAAs are also known to serve as substrates contributing to energy metabolism, expanding the pool of TCA cycle intermediates and gluconeogenesis (25). One study demonstrated that high nitric oxide expenditure (an airway inflammation marker) correlates with increased BCAA levels (13); however, the mechanism behind this remains unknown. The increase in BCAA levels and the relative decrease in BCAA catabolic intermediates could indicate a switch in energetics towards anabolic processes. Thus, these data support the hypothesis that higher doses of inhaled GCs lead to a switch away from TCA cycle-generated ATP. The notion that inhaled GCs affect energy metabolism is further supported by the increase seen in 3-phosphoglycerate and the decreases in TCA cycle intermediates, citrate and aconitate. This metabolomic signature could support a switch in energetics, possibly towards glycolysis. This is in contrast to results from Xu *et al.*, who observed decreased dependence on glycolysis with inhaled GCs, and more efficient oxidative phosphorylation (26). Another study by Bordag *et al.* on the effects of oral dexamethasone on metabolomic signatures in healthy volunteers showed increased gluconeogenesis and reduced glucose utilisation following a single dose (7). Bordag *et al.* found that pyruvate was significantly increased at all timepoints, possibly supporting increased glycolysis (7). However, we did not replicate these findings, with no significant increase in pyruvate seen across the groups. As such, it may be that this decrease in TCA cycle intermediates simply indicates that treatment with inhaled GCs leads to reduced energy expenditure. Importantly, energy expenditure is higher in paediatric patients with asthma (27). Overall, of the molecules studied, BUD induced the greatest systemic changes, which is in agreement with its greater systemic bioavailability (16, 22).

Subjects who received escalating doses of BUD exhibited significantly lower levels of 3-methoxytyramine sulfate (dopamine metabolite) and dopamine 3-*O*-sulfate (regioisomer of dopamine) (17, 18). These data suggest that high doses of inhaled GCs have the potential to alter dopaminergic responses. This is in agreement with anxiety and depression being listed as uncommon side effects of BUD (28). GC therapies have long been associated with psychiatric complications, including depression and mania (29); with depression also recognised as a side effect of adrenal insufficiency (30). Despite these data and our findings, Hyun *et al.* found that inhaled treatments containing GCs had no significant effect on mood among new patients with chronic obstructive pulmonary disease (31).

Altered secondary bile acid metabolites were observed, particularly for BUD and FF. Secondary bile acids are produced in the colon by bacteria metabolising primary bile acids (32). The changes observed, possibly due to the swallowed dose following inhaled GC use, suggest an effect on the microbiome and, potentially, intestinal inflammation (33).

Increases in plasma and TAG and DAG species as doses increase may indicate that inhaled GCs modulate TAG metabolism. GCs can regulate both TAG synthesis and hydrolysis depending on physiological context (34). Once activated by cortisol or another GC, GC receptor binds and positively regulates the transcription of two genes involved in TAG synthesis: glycerol-3-phosphate acyl transferase 3 (*GPAT3*) and lipin 1 (*LPIN1*) (34, 35). The protein encoded by GPAT3 converts glycerol-3-phosphate to lysophosphatidic acid (17). LPIN1 protein dephosphorylates phosphatidic acid to yield DAG (17). In general, the liver, intestine and adipose tissue represent the primary locations for triglyceride synthesis (36, 37). Since TAG synthesis represents an anabolic pathway and a physiological strategy to store fatty acids for later energy consumption (36), drug-induced changes in TAG handling may indicate alterations to systemic energy supply and demands or preference for a particular energy source (e.g. glucose vs fatty acids) and moreover is a mechanism by which chronic high exposure to GCs can cause weight gain and fat redistribution.

All treatments (including placebo) were associated with increases in TAG plasma levels; however, FP showed the highest increase in TAG and DAG levels and significant decreases in FFAs. The greatest number of TAG/DAG/FFA metabolites become differentially enriched/depleted during the first 2–3 weeks of treatment, which may indicate an initial switch in energy demands/preference that later reverts as treatment continues, perhaps due to physiological adaptation to the inhaled GCs. This change in TAG synthesis coincides with a potential decrease in TCA cycle intermediates, which could indicate reduced fatty acid beta-oxidation and a switch towards another energy source. Importantly, increased resting energy expenditure has been reported in childhood asthma (27) and increased fatty acid oxidation has been reported in airway epithelium from mouse models of asthma (38), so perhaps this increase in TAG synthesis indicates a switch to reduced energy expenditure (27).

The focus of our study was inhaled GC molecules that are delivered directly to, and to some extent are retained within, the pulmonary tissue, with low oral bioavailability of the swallowed dose, resulting in lower systemic absorption compared with oral or parenteral GC dosing routes (22). Hence, inhaled GC therapy is expected to be associated with less systemic activity compared to orally administered GCs. Although no within-study comparison of inhaled and oral GC metabolic effects has been reported, orally administered dexamethasone (4000 μg single dose) has been shown to induce large changes in the metabolome, deregulating 150/214 metabolites (7). Several systems were affected by oral GC treatment, including suppression of endogenous steroid hormone production; interruption of circadian rhythm; redisposition of the main energy pathways; and modulation of inflammation mediators and precursors. Although qualitatively these changes are similar to those we found following inhaled GCs, the magnitude of the changes was far greater. This is expected since, compared to inhaled GC therapy, the dose of oral dexamethasone was higher (4000 μg), as was its bioavailability (≈80% (39)) but with lower clearance, resulting in higher systemic exposure.

This study had several limitations. Global metabolomics is a comprehensive method for identifying changes in pathophysiological states; however, as false positives can arise from multiple testing, we used q-values (as a measure of the false discovery rate) in the metabolomic data for every metabolite comparison. Furthermore, our use of the minimum observed value to impute missing values assumed that these values were missing due to the metabolite being below the limit of detection, meaning that the values were missing not at random (MNAR). Our approach is appropriate for MNAR values; however, if most missing values were missing for another reason, a different method might have been more appropriate (40). It is possible that some environmental factors could have affected the findings, for example, while subjects fasted prior to sample collection and were asked to avoid certain foods, this short-term study was not designed to investigate long-term aspects of diet such as BMI and energy expenditure. Efforts were made to control for environmental factors – for example, the time of clinic admission and the timing of metabolomic sample collection – which may not have coincided with maximum metabolic changes. A single 12-h measurement lacks sensitivity compared to 24-h profiling, and the 12-h timepoint employed may have missed effects at earlier timepoints. By including measurements at other timepoints, a greater effect on the metabolome may have been detected and, with serial sampling, a profile of metabolic effects over time could be described; however, this was not possible in this analysis. That said, the sample was deemed to be at a steady state after 7 days of dosing. Additionally, cortisol levels vary widely during the day due to pulsatile secretion and diurnal rhythm, which may have led to discrepancies in cortisol readings between single timepoint metabolomic samples and full 24-h plasma measurements. Notably, cortisol levels did not change much, particularly at lower doses, based on the metabolomic analysis. Whereas, the dose-related effects of the inhaled GCs on DHEA-S were well defined, which is consistent with its longer half-life and less variable plasma concentration. Despite study participants being advised regarding managing environmental factors, these were difficult to control between clinic visits. However, analysis over a wide range of doses for three different inhaled GCs strengthens our conclusions, particularly where evidence of a dose–response was found. Additionally, any potential site- or country-specific factors were thought to be negligible because the majority of subjects randomised were from the UK (82%) and evenly distributed between the two UK centres.

Overall, widespread and in some cases dose-dependent metabolomic changes occurred with inhaled GCs but larger (> 20%) and statistically significant (*P* ≤ 0.05) changes were seen predominately at high and supratherapeutic doses. The most sensitive and dose-responsive markers of exogenous GC systemic exposure were the glucuronide metabolites of cortisol and cortisone and DHEA-S. Of note, qualitative decreases in dopamine metabolites were seen only with BUD. Our results support the safety of low/mid inhaled GC therapeutic doses. However, it is notable that, at comparable therapeutic doses, FF (the inhaled GC with the highest topical potency, greatest lung retention, lowest therapeutic dose and lowest systemic exposure) at 100 µg once daily had the least effect on adrenal steroid pathways, compared with FP and BUD. The risk of systemic effects of inhaled GCs should be considered in the context of their therapeutic benefits.

## Supplementary Material

Supplementary Material

## Declaration of interest

P D-Y, N Br, S S and N Ba are employees of GSK and hold stock/shares in the company. B K is an employee of Metabolon, Inc., which received funding from GSK to conduct the study. D S has received personal fees from Aerogen, AstraZeneca, Boehringer Ingelheim, Chiesi, Cipla, CSL Behring, Epiendo, Genentech, GSK, Glenmark, Gossamerbio, Kinaset, Menarini, Novartis, Pulmatrix, Sanofi, Synairgen, Teva, Theravance and Verona.

## Funding

This study was sponsored and funded by GSK (study 203162).

## Data sharing statement

Information on GSK’s data sharing commitments and requesting access to anonymised individual participant data and associated documents can be found at www.clinicalstudydatarequest.com.
