# Supplementary material for: Inhaled glucocorticoid-induced metabolome changes in asthma
Source: Eur J Endocrinol. 2022 Jul 4;187(3):413–27. doi: 10.1530/EJE-21-0912 (PMC9346266; doi:10.1530/EJE-21-0912)
Supplement: Supplementary Material [file supplementary_material.pdf]

Inhaled glucocorticoid-induced metabolome changes in asthma – Supplementary Materials

Short title: Inhaled glucocorticoids and altered metabolome

Peter Daley-Yates[1], Brian Keppler[2], Noushin Brealey[3], Shaila Shabbir[3], Dave Singh[4] and Neil Barnes[5,6]

[1]Clinical Pharmacology and Experimental Medicine, GlaxoSmithKline plc., Uxbridge, UK, [2]Discovery and Translational Sciences, Metabolon Inc., Morrisville, North Carolina, USA, [3]Medicines Research Centre, GlaxoSmithKline plc., Stevenage, UK, [4]University of Manchester, Medicines Evaluation Unit, Manchester University NHS Foundation Trust, Manchester, UK, [5]Global Medical Franchise, GlaxoSmithKline plc., Brentford, UK, [6]William Harvey Institute, Bart's and the London School of Medicine and Dentistry, London, UK

SUPPLEMENTARY METHODS

Metabolomic profiling was performed as described previously (Ford *et al*, 2020). An overview of the process is provided below.

Sample Preparation

Samples were prepared using the automated MicroLab STAR® system from Hamilton Company. Several recovery standards were added prior to the first step in the extraction process for QC purposes. Samples (100 µL) were extracted with methanol (500 µL) under vigorous shaking for 2 min (Glen Mills GenoGrinder 2000) to precipitate the protein and dissociate small molecules bound to the protein or trapped in the precipitated protein matrix, followed by centrifugation (10 minutes at 680*g*) to recover chemically diverse metabolites. The resulting extract was divided into five fractions: two for analysis by two separate reverse phase (RP)/UPLC-MS/MS methods using positive ion mode electrospray ionization (ESI), one for analysis by RP/UPLC-MS/MS using negative ion mode ESI, one for analysis by HILIC/UPLC-MS/MS using negative ion mode ESI, and one reserved for backup. Samples were placed briefly on a TurboVap® (Zymark) to remove the organic solvent. The sample extracts were stored overnight under nitrogen before preparation for analysis.

Quality Assurance/Quality Control

The following mitigations were used to ensure quality assurance (QA) and quality control (QC) between samples: 1) technical replicate samples derived from a pool of well-characterized human plasma (MTRX) ~~or, alternatively, generated by combining a small portion of each (non-plasma) experimental sample (CMTRX),~~ were spaced evenly among experimental samples; 2) extracted water samples (process blanks) and solvent blanks; and 3) a cocktail of QC standards, were carefully chosen so as not to interfere with the measurement of endogenous compounds, and were spiked into every analyzed sample, allowing instrument performance monitoring and aiding with chromatographic alignment.

Ultrahigh Performance Liquid Chromatography-Tandem Mass Spectroscopy (UPLC-MS/MS)

All methods utilized a Waters ACQUITY ultra-performance liquid chromatography (UPLC) and a Thermo Scientific Q-Exactive high resolution/accurate mass spectrometer interfaced with a heated electrospray ionization (HESI-II) source and Orbitrap mass analyzer operated at 35,000 mass resolution. The sample extract was dried then reconstituted in solvents compatible with each of the four methods. Each reconstitution solvent contained a series of standards (isotopically labeled or halogenated compounds; see Supplementary Table 1) at fixed concentrations to ensure injection and chromatographic consistency, and for alignment during data processing. One aliquot was analyzed using acidic positive ion conditions, chromatographically optimized for more hydrophilic compounds. In this method, the extract was eluted from a C18 column (Waters UPLC BEH C18-2.1x100 mm, 1.7 µm) using a gradient consisting of water and methanol, containing 0.05% perfluoropentanoic acid (PFPA) and 0.1% formic acid (FA). A second aliquot was also analyzed using acidic positive ion conditions, and was chromatographically optimized for more hydrophobic compounds. In this method, the extract was eluted over a gradient of methanol, acetonitrile, water, 0.05% PFPA and 0.01% FA, and was operated using an overall higher organic content. A third aliquot was analyzed using negative ionization optimized conditions using a separate dedicated C18 column. The basic extracts were eluted using a gradient of methanol and water with 6.5 mM ammonium bicarbonate at pH 8. The fourth aliquot was analyzed via negative ionization following elution from a

HILIC column (Waters Acquity UPLC BEH Amide 2.1 x 150 mm, 1.7 µm) using a gradient consisting of water and acetonitrile with 10 mM ammonium formate, overall pH 10.8 with ammonium hydroxide. The MS analysis alternated between MS and data-dependent MS^n scans using dynamic exclusion. The scan range varies slightly between methods, but covers approximately 70-1000 m/z. Raw data files are archived and extracted as described below.

Reference

Ford L, Kennedy AD, Goodman KD, Pappan KL, Evans AM, Miller LAD, Wulff JE, Wiggs BR, Lennon JJ, Elsea S, Toal DR. Precision of a Clinical Metabolomics Profiling Platform for Use in the Identification of Inborn Errors of Metabolism. *J Appl Lab Med*. **2020** Mar 1;5(2):342-356.

Trademark statement

All trademarks are the property of, or licenced to, their respective owners (Hamilton Company and Zymark).

Supplemental Table 1. Chromatography Conditions

	Chromatographic Conditions			
	**LC/MS Positive (positive ionization chromatography optimized for hydrophilic compounds)**	**LC/MS Positive (positive ionization chromatography optimized for hydrophobic compounds)**	**LC/MS Negative (negative ionization optimized conditions)**	**LC/MS Negative (negative ionization with HILIC chromatography)**
Column	Waters BEH C18 1.7µm 2.1 x 100mm	Waters BEH C18 1.7µm 2.1 x 100mm	Waters BEH C18 1.7um 2.1 x 100mm	Waters BEH C18 1.7µm 2.1 x 150mm
Mobile Phase A	0.1% formic acid and 0.05% PFPA in water, pH ~2.5	0.1% formic acid and 0.05% PFPA in water, pH ~2.5	6.5 mM ammonium bicarbonate in water, pH 8	10 mM ammonium formate in 15% water/5% methanol/80% acetonitrile (effective pH 10.16 with NH_4OH)
Mobile Phase B	0.1% formic acid and 0.05% PFPA in methanol, pH ~2.5	0.1% formic acid and 0.05% PFPA in 50% methanol/ 50% acetonitrile, pH ~2.5	6.5 mM ammonium bicarbonate in 95% methanol/5% water	10 mM ammonium formate in 50% water/50% acetonitrile (effective pH 10.60 with NH_4OH)
Flow Rate	0.35 mL/min	0.60 mL/min	0.35 mL/min	0.50 mL/min
Gradient Elution	Linear gradient from 5% B to 80% B over 3.35 minutes	Linear gradient from 40% B to 99.5% B over 1.0 minute, hold 99.5% B for 2.4 minutes.	Linear gradient from 0.5 to 70% B over 4.0 minutes, then rapid gradient to 99% B in 0.5 minutes.	Linear gradient from 5% B to 50% B in 3.5 minutes, then linear gradient from 50% B to 95% B in 2 minutes.
Instrument Performance Standards	d7-glucose	Br-phenylalanine	d7-glucose	d35-octadecanoic acid
	d5-glutamine	d5-androstene	d3-methionine	d5-indole acetate
	d2-threonine	d9-progesterone	d3-leucine	Br-phenylalanine
	d5-hippuric acid	d4-dioctyphthalate	d8-phenyulalanine	d5-tryptophan

	d3-methionine		d5-tryptophan	d4-tyrosine
	d3-leucine		Br-phenylalanine	d3-serine
	Br-phenylalanine		d15-octanoic acid	d3-aspartic acid
			d19-decanoic acid	d7-ornithine
			d27-tetradecanoic acid	d4-lysine
			d35-octadecanoic acid	
			d2-eicosanoic acid	
Process Assessment Standards	fluorophenylglycine	d6-cholesterol	tridecanoic acid	fluorophenylglycine
	chlorophenylalanine	chlorophenylalanine	chlorophenylalanine	chlorophenylalanine

BEH, ethylene bridged hybrid; LC, liquid chromatography; MS, mass spectrometry; PFPA, perfluoropentanoic acid
